# Prevalence and Correlates of Likely Major Depressive Disorder among Medical Students in Alberta, Canada

**DOI:** 10.3390/ijerph191811496

**Published:** 2022-09-13

**Authors:** Sumeet Dhanoa, Folajinmi Oluwasina, Reham Shalaby, Esther Kim, Belinda Agyapong, Marianne Hrabok, Ejemai Eboreime, Maryana Kravtsenyuk, Alicia Yang, Izu Nwachukwu, Chantal Moreau, Adam Abba-Aji, Daniel Li, Vincent I. O. Agyapong

**Affiliations:** 1 Department of Psychiatry, University of Alberta, Edmonton, AB T6G 2R3, Canada; 2 Department of Psychiatry, University of British Columbia, Vancouver Island, BC V6T 1Z4, Canada; 3 Alberta Health Services, Edmonton, AB T5J 3E4, Canada; 4 Department of Psychiatry, University of Calgary, Calgary, AB T2N 1N4, Canada; 5 QEII Health Sciences Centre, Department of Psychiatry, Faculty of Medicine, Dalhousie University, 5909 Veterans Memorial Lane, 8th Floor, Abbie J. Lane Memorial Building, Halifax, NS B3H 2E2, Canada

**Keywords:** major depressive disorder, social support, prevalence, medical students

## Abstract

Background: Medical students are exposed to multiple factors during their academic and clinical studies that contribute to depression. Aims: This study aims to examine the prevalence and correlates of likely major depressive disorder (MDD) among medical students. Methods: This study utilized a descriptive cross-sectional design. Data were collected through a self-administered online survey, which included questions on sociodemographic characteristics and likely MDD using the PHQ-9. Data were analyzed using a descriptive, Chi-square test and logistic regression model. Results: There were 246 medical students who participated in the survey. The majority were females, 155 (65.1%); Caucasian, 158 (66.4%); and in a relationship, 168 (70.5%). The prevalence of likely MDD was 29.1%. Respondents who did not feel supported and respondents who were neutral about their social support, friends, and family, were 11 and 4 times more likely to experience MDD than those who felt well supported (OR = 11.14; 95% CI: 1.14–108.80) and (OR = 4.65; 95% CI: 1.10–19.56), respectively. Conclusions: This study suggests a high prevalence of likely MDD among medical students who do not feel they have sufficient social support from friends and family. Social adjustments, including talking to friends and family and participating in leisure activities, could reduce the level of depression among medical students.

## 1. Introduction

Medical schools train intelligent and altruistic individuals with a strong commitment to practice patient care and promote public health despite the significant demands and challenges associated with the medical profession [1]. Although the education process is anticipated to nurture students’ personal growth and well-being, current studies suggest a high frequency of depression and anxiety among medical students [1,2,3,4]. Canadian medical education is rigorous in nature [1] and can be a time of great distress for physicians in training, potentially leading to inadvertent negative impacts on students’ mental health, academic performance, and professionalism [1,2,3,4].

The criteria for major depressive disorder (MDD) are well defined according to the Diagnostic and Statistical Manual, Fifth Edition (DSM-V). In the remainder of the paper, the term depression is used as a proxy for MDD.

Depression is a pervasive and common mental disorder throughout the world. According to the World Health Organization, about 4.4% of the global population, or 322 million people, were living with depression in 2015 [5]. In the US general population, the Composite International Diagnostic Interview (CIDI) scale estimated that the lifetime prevalence of MDD was 16.2% and the 12-month prevalence was 6.6% [6] based on household surveys conducted from February 2001 to December 2002. In Canada, the prevalence of MDD in 2012 was 3.9% [7].

The medical student population is not immune to depression. Several systematic reviews and cross-sectional institutional studies have explored the prevalence of depression among medical students using various instruments. Commonly used instruments include the Primary Care Evaluation of Mental Disorders (PRIME-MD) [8], Patient Health Questionnaire (PHQ-9) as the self-administered version of PRIME-MD [9], the Beck’s Depression Inventory [10], Major Depression Inventory [11], and the 12-item General Health Questionnaire [12]. Dyrbye et al. reported that there was a higher level of psychological distress (including depression, anxiety, burnout, and related mental health problems) and a lower quality of life among US and Canadian medical students compared to both the general population and age-matched peers [1,13,14]. In keeping with these findings, a higher prevalence of depression among medical students has been reported in other parts of the world [15,16,17,18,19,20]. Globally, the mean prevalence of depression among medical students was estimated at 28% according to a 2016 meta-analysis [2]. Other systematic reviews have also estimated prevalence rates upwards of 28% [3,4].

It is unclear whether medical students have a greater predisposition to depression at the beginning of their training compared to students in other educational streams [1]. A cross-sectional study of six US medical schools found that medical students at the beginning of medical school had lower rates of depression symptoms (26.2%) compared to their age-matched college graduates (42.4%) [21]. These findings, when considered along with the high prevalence of depression among medical students, suggest that the training environment could potentially contribute to a decline in students’ mental health.

There are several correlational factors contributing to depression among medical students according to various studies. The individual factors associated with higher odds of depression symptoms among medical students include being of the female sex [1,14,17,19,22,23,24], a younger age [23], poor physical health [25], low levels of perceived interpersonal support [17,26], interpersonal conflicts between students and teachers largely in informal settings [27], personal life events (such as the death of a family member, personal illness or injury, etc.) [27], financial insecurity [17,28], educational debt [27], poor quality of sleep and an irregular diet [26], dissatisfaction with education [16], low socioeconomic status [16], family history of depression [16], and emotional abuse outside school [29]. Mixed results have been reported about the relationship between depression among medical students and ethnicity [23].

In terms of the relative association of these factors with MDD, a cross-sectional study of 1068 Mexican medical students reported that current emotional abuse outside school was most strongly associated with MDD, followed by a personal history of depression, a family history of depression, and perceived academic stress [29]. On the other hand, being married was associated with lower odds of having depression symptoms [14].

The environmental factors associated with higher odds of depression symptoms among medical students include an earlier stage of medical training [2,14,20,23,30], adverse medical school environment [25], competitive nature of medical education [4,19], adjustment to the demanding training environment [14,27], academic overload [17,27], exposure to death and suffering through clinical encounters [27], insufficient preparedness to confront issues related to death and dying due to lack of education about end-of-life issues and palliative care [27], and student abuse (including verbal abuse, physical abuse, sexual harassment, and racial discrimination) and were relatively more prominent among female students [27]. In terms of the stage of medical training, it is worth noting that a systematic review of 195 studies involving 129,123 medical students in 47 countries found no statistically significant difference in depression prevalence between preclinical and clinical students [4].

Depression among medical students has been associated with several potential adverse outcomes including impaired academic performance in both preclinical and clinical years, a decline in empathy and compassion that ultimately erodes professionalism, and a loss of academic integrity [17,27]. Distressed medical students are more likely to quit medical school despite their good academic standing [1]. They are also more likely to develop unhealthy coping mechanisms including consuming excessive alcohol [1] and engaging in substance use [30].

In the long-term, the relatively high rates of depression among medical students can have negative implications for physician health and the quality of patient care [4]. Individuals may struggle with a low sense of personal accomplishment and may not be able to play meaningful roles in providing patient care [14]. What exacerbates this further is the higher likelihood of medical students with depressive symptoms endorsing stigmatized attitudes towards depression [24]. This may result in medical students’ reluctance to seek treatment for depression [20], as doing so may jeopardize their careers [31] or invoke perceptions of lower intelligence [24].

There is a lack of extensive research on potential interventions that could help mitigate depression among medical students [1]. Social support from friends, family, peers, and faculty is a well-established protective mechanism against depression [32,33,34,35]. Other helpful coping mechanisms include counselling services and extracurricular activities [36]. At the individual level, resilience has been shown to potentially moderate depression and can work against it even in the presence of academic burnout [37]. At a systemic level, it has been suggested that interventions aimed at reducing the prevalence of depression should be delivered in medical school so that doctors are made aware of how to access appropriate support [38].

Lastly, there is a dearth of Canadian studies related to the prevalence and correlates of likely MDD among medical students. A 1997 study conducted at McGill University in Ontario found elevated depression scores in a minority of medical students during the transition to clinical training [39]. A 2015–2016 survey of medical students in all years of study at all 17 Canadian medical schools estimated that major depression in Canadian medical students was at 18.1%, concluding that medical students aged 20–34 had significantly higher rates of diagnosed depression, diagnosed anxiety disorders, suicidal ideations, and psychological distress compared to the age-matched general population of postsecondary graduates [40]. In addition to the insufficient Canadian data, there is a lack of provincial data regarding depression rates and correlates. It is important to explore this issue in provincial contexts, as healthcare is largely delivered provincially in Canada.

The goal of the current study is to evaluate the prevalence of likely MDD in medical students in Alberta and identify the associated individual and environmental factors. It is hypothesized that the prevalence findings will be consistent with those reported in other jurisdictions. The results of this study concerning the correlational factors will inform the development of interventions and preventative measures aimed at reducing depression rates among medical students.

## 2. Methods

### 2.1. Study Setting and Design

This study was conducted in Alberta, a western province of Canada [41]. As of December 2018, there were 1148 medical students registered with the College of Physicians and Surgeons of Alberta, the provincial regulatory body [42].

This study utilized a descriptive cross-sectional study design through self-administered, anonymous, online questionnaires. At the time of this study, the total medical student population in Alberta was 1148; an anticipated sample size of 777 was determined based upon a 95% confidence level and a margin of error of 2% for the prevalence rate estimates for medical students’ burnout. However, the total respondents were 177 medical students attending the University of Alberta’s Faculty of Medicine and Dentistry, and the University of Calgary’s Cumming School of Medicine.

### 2.2. Institutional Review Board Approval and Participants’ Consent

The study was conducted following the Declaration of Helsinki (Hong Kong Amendment) and Good Clinical Practice (Canadian Guidelines). All participants were provided with an online information leaflet, and informed consent was obtained before participation following the Declaration of Helsinki. The study received ethical approval from the Health Ethics Research Board of the University of Alberta (reference number Pro00091436) and the Conjoint Health Research Ethics Board of the University of Calgary (REB19-1457).

### 2.3. Data Collection Tool

Data collection tools for this study were developed based on the published literature and questions from previously validated instruments. Likely major depressive disorder (MDD) was assessed using the cut score of ≥10 on the Patient Health Questionnaire-9 (PHQ-9 [9,43]. These standardized measures provided information for the study results. In addition, the survey included an open-ended question to facilitate qualitative data collection. Kroenke et al., 2001, established the diagnostic validity of the 9-item PHQ-9 scores > 10 to have a sensitivity of 88% and a specificity of 88% for major depressive disorder. The internal consistency of the PHQ-9 has been shown to be high with Cronbach alphas of 0.86 and 0.89, respectively, from two different patient populations [9]. The sections of the survey questionnaire included in this paper are published in Appendix A.

### 2.4. Statistical Analysis

Data were analyzed using SPSS (Version 26; IBM Corp, Armonk, NY, USA) [44]. The data were presented as absolute numbers and percentages according to gender for all the demographic and clinical variables. The Chi-square test/Fisher’s exact analysis with two-tailed significance (*p* ≤ 0.05) was performed to assess the association between the sociodemographic and perceived interprofessional variables and likely MDD in the medical students as measured using the PHQ-9. Variables with statistically significant or near-significant associations (*p* ≤ 0.1) for each of the variables were entered into a logistic regression model to identify significant predictors of likely MDD. Before performing the logistic regression analysis, correlational diagnostics were performed to identify any strong inter-correlations (Spearman’s correlation coefficient from 0.7 to 1.0 or −0.7 to −1.0) among the predictor variables. Odds ratios from the binary logistic regression analysis were calculated to determine the association between the predictor variables and likely MDD.

## 3. Results

Of the 1148 medical students reached in Alberta with the survey link, there were 272 responses received, giving a response rate of 23.7%. Out of the 272 responses received, 246 contained at least partially completed responses, 177 contained fully completed responses, and 26 contained responses that were left blank. Only partially and fully completed responses were included in the analysis, giving an effective response rate of 21.4%.

Table 1 shows the distribution of the sociodemographic information as well as the academic background of the respondents.

Of the medical students who participated in the survey, the majority, 65.1% (155), were female and 34.9% (83) were male. Again, 32.4% (77) of the respondents were aged under 25, 53.4% (127) were aged between 25 and 30 years, and 13.4% (32) were aged 31–40 years. Only two (0.8%) of the respondents were aged 40–60 years.

This study revealed that the predominant ethnicity of respondents was Caucasian at 66.4% (158), whereas 16.4% (39) were Asian, and the lowest proportion, 17.2% (41), were others (Indigenous and Black people).

Overall, 29.3% (76), 29.7% (77), and 19.7% (51) of respondents were in their first, second, and third years, respectively. Most of the respondents, 70.5% (168), were in a relationship and the remaining 29.5% (70) were single.

Table 1 shows the summary statistics of professional satisfaction, workplace collegiality, and support. Most, 77.4% (161), were satisfied with the quality of peer collaboration with their colleagues. A majority, 66.7% (138), of respondents reported that they were satisfied with their quality of interaction with physicians and residents, whereas 12.1% (25) were dissatisfied.

The majority of the respondents were satisfied with the professional relationships they had with their colleagues. Although control and flexibility are quite relevant to the training and development of healthcare providers, including medical students, nearly half, 45.4% (94), of the respondents agreed that they were satisfied with the flexibility in their profession, 33.3% (69) were dissatisfied, and 21.3% (44) were neither satisfied nor dissatisfied.

Eighty-six per cent (175) agreed that they found their colleagues to be very supportive, whereas very few, 6.9% (14), disagreed with the fact that their colleagues were not supportive. Significantly, 89.2% (182) of respondents agreed that people treated each other with respect and 4.9% (10) disagreed that people treated each other with respect. Nearly half, 48% (86), of the respondents reported that their medical schools had enough strategies aimed at students’ well-being.

Table 2 shows a summary of the results of a univariate analysis of the association between respondents’ demographic profiles, professional satisfaction, and workplace collegiality variables and major depressive disorder (MDD).

The Chi-square/Fisher’s exact test showed that only 11 sociodemographics, professional satisfaction, and workplace collegiality variables have a statistically significant association with likely MDD. These variables include ethnicity; the quality of the learning environment; the workload and job demand, control, and flexibility; work–life integration; efficiency and resources; satisfaction with pursuing a career in medicine; the level of support from colleagues; how well-supported they felt by their social support/friends and family; what would occur if they reached out for help to those in their learning and work environments; and medical schools having enough strategies aimed at medical students’ well-being in place.

Table 3 below shows the logistic regression model to determine the independent predictive variables for likely MDD among the medical students who participated in the survey. The model included 11 significant predictor variables and three other variables that showed near significant associations with likely MDD in the Chi-square/Fisher’s exact test. None of the variables showed a high correlation (r_s_ > 0.7) with another variable.

The logistic regression model was statistically significant; *Χ*^2^ (df = 26; *n* = 177) = 63.97, *p* < 0.001, indicating that the model could differentiate between study participants with the presence and absence of likely MDD. The model explained 30.3% (Cox and Snell R^2^) to 43.2% (Nagelkerke R^2^) of the variance. According to the goodness-of-fit statistic using the Hosmer–Lemeshow goodness-of-fit test, the model was adequately fit (Chi^2^ = 12.30; *p* = 0.91) and correctly classified 83.6% of cases.

Table 3 shows that only one variable, *how well the medical students felt supported by their social support/friends/family,* independently predicted the likely presence of MDD in respondents.

With all other variables controlled for in the regression model, respondents who did not feel supported by their social support, friends, and family were 11 times more likely to experience MDD than those who felt well supported (OR = 11.14; 95% CI: 1.14–108.80). On the other hand, respondents who were neutral about being supported by friends and family were four times more likely to experience MDD than respondents who reported that they felt very well supported by their friends and family (OR = 4.65; 95% CI: 1.10–19.56).

## 4. Discussion

The current study evaluated the prevalence of depression among medical students in Alberta by surveying students from both medical schools in the province. It also explored the potential correlations of depression with various individual and environmental factors including the demographics, quality of the learning environment, workload, resources, satisfaction level, work–life balance, social support, and support from medical school. To our knowledge, this is the first study to examine the prevalence of MDD and the potential correlates among medical students in Alberta.

### 4.1. Depression Prevalence

Our study found that the mean prevalence of likely MDD among medical students in Alberta was 29.1%. This finding is consistent with the prevalence of depression (28%) estimated among medical students globally [2,3,4]. The prevalence of depression among medical students in the UK, Europe, and the wider Anglophone world, excluding North America, ranged from 6% to 66% [15]. A systematic review involving 35,160 Chinese medical students estimated that the mean prevalence of depression was 32.74% [16]. Another systematic review of 25 studies investigating depression in medical students in Brazil reported a prevalence rate of 30.6% [17]. A 2008 survey of 120 female medical students in Seoul, Korea, found a depression rate of 37.1% using the Center for Epidemiology Studies Depression Scale. A cross-sectional study performed in 12 medical schools in Italy found the prevalence of depression among medical students was 29.5% [18] using the Beck Depression Inventory-II [10]. A cross-sectional study of 342 medical students in a Swedish university reported a depression prevalence of 12.9% using the Major Depression Inventory (MDI) [11,19]. A cross-sectional study of 237 medical students in an Indian university deploying the Patient Health Questionnaire (PHQ-9), based on the Primary Care Evaluation of Mental Disorders, reported a 21.5% prevalence of provisionally diagnosed depressive disorder and a 7.6% prevalence of MDD among medical students [20].

A relatively lower prevalence of depression at 11% was found among Asian medical students by a systematic review involving 10,147 students [30]. The authors hypothesized that this relatively low pooled prevalence compared to other parts of the world could be attributed to underreporting as some Asian cultures do not view mental health issues favourably, medical students’ self-psychological management due to knowledge of psychiatry and medical psychology, and medical schools’ support for mental health [30].

Some medical students have experienced deteriorated mental health during the COVID-19 pandemic. A February 2020 survey of 5982 medical students in China found a relatively higher rate of depression of 35.2%.

### 4.2. Depression Correlates

Our study found that the lack of social support independently predicted the likely presence of MDD in medical students in Alberta. Respondents who did not feel supported by their social support, friends, and family were 11 times more likely to experience MDD than those who felt well supported. Respondents who were neutral about being supported by social support/friends and family were four times more likely to experience MDD than respondents who reported that they felt very well supported by their friends and family. Conversely, the presence of social support predicted the likely absence of MDD.

Social support is a multidimensional concept that refers to “the psychological and material resources available to individuals through their interpersonal relationships” [45]. A clear association between social support and depression has been established. Although actual or perceived social support may positively influence mental health, its absence appears to contribute to the onset of depressive symptoms [46,47].

Overall, this study suggests a high prevalence of likely MDD among medical students who do not feel they have sufficient social support from friends and family. This aligns with findings from other studies that inadequate social activities or support resulted in the decreased psychological health of medical students [26,48,49,50,51]. For example, Jeong et al. found that medical students with lower levels of interpersonal support were ten times more likely to suffer from depression [26]. Furthermore, a lack of social support is associated with an increased risk of burnout including emotional exhaustion and a sense of low personal accomplishment [50].

Social support from friends, family, peers, and faculty has been shown to protect medical students from depression [32,33,34,35]. Family, in particular, is a key source of support for medical students and hence a negative relationship was found between family dynamics and depression [51]. A six-year longitudinal study by Kjeldstadli et al. also indicated that medical students who perceived medical school as interfering less with their social and personal lives were psychologically more stable and sustained high levels of life satisfaction [52]. In addition, in a February 2020 survey of 5982 medical students in China, medical students with a low level of social support were at an approximately three times higher risk of depressive or anxiety symptoms than those with a high level of social support [35]. This highlights the increased importance of improving the social support of medical students during the pandemic. Seeking social support was also correlated with positive emotions in medical students including maintaining empathy among medical students [53], which is essential for their future professional roles as physicians. This underscores the importance of designing interventions aimed at strengthening social support among medical students.

In view of our findings affirming the benefits of social support in alleviating depression, medical students should be encouraged to engage in social activities both within and outside medical school. Social adjustments, including talking to friends and family and participating in leisure activities, could reduce the levels of depression. Canadian medical schools have incorporated several faculty and student-run mental health initiatives such as mindfulness workshops, meditation and yoga workshops, wellness blogs, social gatherings, and peer-support programs, to name a few [54]. Medical school educators should continue to play a role in facilitating social interactions among medical students. A culture focused on student and practitioner well-being, self-compassion, and self-care could potentially play a significant role in reducing depression rates. In addition, other interventions such as counselling and mental health services [36], extracurricular activities [36], and emotional resilience skills training [55] could improve students’ well-being. Demographic, professional satisfaction, and workplace collegiality variables did not independently predict likely MDD among the medical students in this study. Thus, unlike other studies that reported higher rates of depression symptoms among female medical students [1,14,17,19,22,23,24] and those of a younger age [23], this study found no such association. Similarly, the environmental factors, such as an earlier stage of medical training [2,14,20,23,30], adverse medical school environment [25], competitive nature of medical education [4,19], adjustment to the demanding training environment [14,27], and academic overload [17,27], which have been found to be associated with higher odds of depression symptoms among medical students in other studies, did not independently predict likely MDD in this study.

### 4.3. Limitations

Our study has some limitations. First, the findings are based on a small sample size. Although the survey was sent to all 1148 medical students in Alberta and a sample size of 777 was projected based upon a 95% confidence level and a margin of error of 2% for likely MDD prevalence, the study achieved a much smaller sample size. Thus, based on a population sample of 1148, an actual sample size of 246, and a 95% confidence interval, the margin of error for the likely MDD prevalence estimates was 6%, which is higher than the projected 2% determined a priori. Second, the study was cross-sectional in nature and therefore lacked the predictive ability of a longitudinal study. Third, the survey-based nature of our study made it prone to response and recall bias; depressed students may be more likely to fill out the survey due to its personal relevance or less likely to fill it out due to a lack of motivation. Fourth, the study was conducted during the global COVID-19 pandemic, which had significant implications for medical education, including an increased reliance on online education and technological tools and subsequently, fewer social interactions among medical students. These unprecedented changes in medical education may have affected the survey responses and consequently the study results. Lastly, several other unmeasured confounding factors could potentially affect the depression rates among medical students in Alberta. These factors may include but are not limited to, the immigration status of medical students, types of social relationships that students engage in, years of education before medical school, years of employment before medical school, socioeconomic status of medical students, and exposure to the medical profession before medical school, to name a few.

More research needs to be conducted on the correlational and causal factors associated with depression among medical students in Canada so that effective mitigation strategies can be developed to help improve their psychological health.

## 5. Conclusions

This study has established a prevalence of likely MDD among medical students in Alberta of 29.1 ± 6%, which is comparable to the prevalence of likely MDD recorded among medical students in other jurisdictions. Social support from friends and family was the only independent predictor for likely MDD. This suggests that social adjustment, including talking to friends and family and participating in leisure activities, could reduce the level of depression among medical students. Canadian medical educators should emphasize the importance of social support when counselling students about mental health so that students are encouraged to participate in social activities in their support network both within and outside medical school. Furthermore, we suggest that more studies need to be conducted to assess the various individual and environmental factors related to depression among medical students in Canada. More research would help delineate the contributory factors and develop effective prevention and mitigation strategies aimed at improving the mental health of medical students.

## Figures and Tables

**Table 1:** (**a**) Sociodemographic characteristics of the respondents. (**b**) Professional satisfaction and workplace collegiality and supports available to respondents.

(a)			
Variable Type	Variable	Frequency (%)	
**Age (years)**	<25	77 (32.4)	
	25–30	12 (53.4)	
	31–40	32 (13.4)	
	40–60	2 (0.8)	
**Gender**	Male	83 (34.9)	
	Female	155 (65.1)	
**Relationship Status**	In a relationship	168 (70.5)	
	Not in a Relationship	70 (29.5)	
**Dependents**	0	233 (90.0)	
	1	15 (5.8)	
	2	9 (3.5)	
	3	2 (0.8)	
**Ethnicity**	Caucasian	158 (66.4)	
	Asian	39 (16.4)	
	Other	41 (17.2)	
**Medical School Year**	First Year	76 (29.3)	
	Second Year	77 (29.7)	
	Third Year	51 (19.7)	
	Fourth Year	29 (11.2)	
	Fifth Year	26 (10.0)	
**(b)**			
**Variable**	**Dissatisfied**	**Neither Satisfied/Dissatisfied**	**Satisfied**
	**N (%)**	**N (%)**	**N (%)**
**Quality of peer collab. among medical students**	23 (11.1)	24 (11.5)	161 (77.4)
**Quality of interaction with physicians and residents**	25 (12.1)	44 (21.3)	138 (66.7)
**Quality of learning environment**	35 (16.9)	41 (19.8)	131 (63.3)
**Workload and job demand**	39 (18.8)	54 (26.1)	114 (55.1)
**Control and flexibility**	69 (33.3)	44 (21.3)	94 (45.4)
**Work–life Integration**	61 (29.5)	57 (27.5)	89 (43.0)
**Efficiency and Resources**	34 (16.4)	74 (35.7)	99 (47.8)
**Variable**	**Disagree**	**Neither Agree nor Disagree**	**Agree**
**Satisfied about pursuing a career in medicine**	13 (6.4)	15 (7.4)	176 (86.3)
**I find colleagues to be supportive**	14 (6.9)	15 (7.4)	175 (85.8)
**People treat each other with respect** **in my work group**	10 (4.9)	12 (5.9)	182 (89.2)
**A spirit of cooperation and teamwork exists in** **my group**	14 (6.9)	17 (8.3)	173 (84.8)
**Disputes or conflicts are resolved fairly in** **my workgroups**	12 (5.9)	51 (25.0)	141 (69.1)
**Variable**	**No**	**Yes**	**Somewhat**
**Medical school strategies aimed at student’s well-being**	36 (20.3)	55 (31.1)	86 (48.6)
**Variable**	**Somewhat well** **N (%)**	**Neutral** **N (%)**	**Somewhat poorly** **N (%)**
**How well do you feel about social support/friends/family?**	154 (87.0)	16 (9.0)	17 (4.0)
**How would you best describe what would occur if you reached out for help to those in your learning and work environments?**	144 (81.4)	22 (12.4)	11 (6.2)

**Table 2:** Association between sociodemographic characteristics, workplace collegiality, and depression.

Characteristics		Number (%)	Chi-square/Fisher’s Exact Test *	Effect Size (Phi/Cramer V’s)	*p*- Value
**Age**	<25	17 (31.5)	2.178	0.110	0.498
	25–30	26 (26.3)			
	31–40	9 (37.5)			
	40–60	0 (0.0)			
**Gender**	Male	19 (36.5)	0.300	0.041	0.584
	Female	33 (63.5)			
**Relationship** **Status**	In a relationship	37 (71.2)	0.001	0.003	0.969
	Not in a relationship	15 (28.8)			
**Dependent**	0	46 (29.1)	2.668	0.122	0.503
	1	3 (23.1)			
	2	2 (28.6)			
	3	1 (100.0)			
**Ethnicity**	Caucasian	29 (55.8)	8.337	0.216	**0.015**
	Asian	10 (19.2)			
	Other	13 (25.0)			
**Current medical school year**	First year	12 (26.7)	5.317	0.172	0.265
	Second year	13 (23.2)			
	Third year	11 (28.9)			
	Fourth year	10 (50.0)			
	Fifth year	6 (30.0)			
**Quality of peer collaboration among med. students**	Dissatisfied	10 (19.2)	5.473	0.175	0.063
	Neither	7 (13.5)			
	Satisfied	35 (67.3)			
**Quality of interaction with your attending physicians and residents**	Dissatisfied	10 (19.2)	5.258 *	0.174	0.067
	Neither	12 (23.1)			
	Satisfied	30 (57.7)			
**Quality of your learning environment**	Dissatisfied	18 (34.6)	21.762	0.349	**0.001**
	Neither	14 (26.9)			
	Satisfied	20 (38.5)			
**Workload and job demand**	Dissatisfied	19 (36.5)	22.689	0.356	**0.001**
	Neither	17 (32.7)			
	Satisfied	16 (30.8)			
**Control and flexibility**	Dissatisfied	32 (5701)	23.765	0.364	**0.001**
	Neither	5 (48.8)			
	Satisfied	15 (13.5)			
**Work–life integration (meeting personal and professional obligations)**	Dissatisfied	28 (53.8)	21.855	0.349	**0.001**
	Neither	13 (25.0)			
	Satisfied	11 (21.2)			
**Efficiency and resources**	Dissatisfied	15 (28.8)	15.218	0.293	**0.001**
	Neither	24 (46.2)			
	Satisfied	13 (25.0)			
**Overall, I am satisfied about pursuing a career in medicine.**	Disagree	6 (11.5)	9.234	0.227	**0.010**
	Neither agree/disagree	8 (15.4)			
	Agree	38 (73.1)			
**In general, I find my colleagues to be supportive.**	Disagree	8 (15.4)	8.157	0.213	**0.013**
	Neither agree/disagree	5 (9.6)			
	Agree	39 (75.5)			
**People treat each other with respect in my work group.**	Disagree	6 (11.5)	0.166	0.213	0.089
	Neither agree/disagree	3 (5.8)			
	Agree	43 (82.7)			
**A spirit of cooperation and teamwork exists in my work group.**	Disagree	6 (11.5)	3.007	0.130	0.231
	Neither agree/disagree	5 (9.6)			
	Agree	41 (78.8)			
**Disputes or conflicts are resolved fairly in my work group.**	Disagree	5 (9.6)	3.656	0.143	0.177
	Neither agree/disagree	17 (32.7)			
	Agree	30 (57.7)			
**How well do you feel supported by your social support/friends/family?**	Somewhat well	38 (73.1)	13.176	0.273	**0.001**
	Neutral	9 (17.3)			
	Somewhat poorly	5 (9.6)			
**How would you best describe what would occur if you reached out for help to those in your learning and work environments?**	Somewhat support.	36 (69.2)	8.963	0.225	**0.010**
	Neutral	9 (17.3)			
	Somewhat hostile	7 (13.5)			
**Do you feel that your medical school has enough strategies aimed at medical students’ well-being in place?**	No	16 (44.4)	19.434	0.331	**<0.001**
	Yes	4 (7.3)			
	Somewhat	32 (37.2)			

* Fishers exact test

**Table 3:** Logistic regression model predicting likely major depressive disorder among the medical students.

Variables in the Equation	B	SE.	Wald	df	*p* Value	Odds Ratio	95% C.I. for Odds Ratio	
							Lower	Upper
**Ethnicity**								
Caucasian			4.493	2	0.106			
Asian	1.391	0.658	4.467	1	0.035	4.018	1.106	14.594
Others	0.284	0.627	0.205	1	0.650	1.329	0.389	4.545
**Quality of peer collaboration among med. students**								
Dissatisfied			2.924	2	0.232			
Neither	0.107	1.014	0.011	1	0.916	1.112	0.152	8.119
Satisfied	−0.997	0.850	1.376	1	0.241	0.369	0.070	1.952
**Quality of interaction with your attending physicians and residents**								
Dissatisfied			1.217	2	0.544			
Neither	−0.656	0.795	0.680	1	0.410	0.519	0.109	2.467
Satisfied	−0.012	0.776	0.000	1	0.987	0.988	0.216	4.519
**Quality of your learning environment**								
Dissatisfied			6.074	2	0.048			
Neither	1.017	0.771	1.739	1	0.187	2.764	0.610	12.532
Satisfied	−0.687	0.682	1.015	1	0.314	0.503	0.132	1.916
**Workload and job demand**								
Dissatisfied			0.205	2	0.903			
Neither	−0.178	0.675	0.069	1	0.793	0.837	0.223	3.144
Satisfied	−0.353	0.783	0.204	1	0.652	0.702	0.151	3.258
**Control and flexibility**								
Dissatisfied			5.766	2	0.056			
Neither	−1.867	0.817	5.220	1	0.022	0.155	0.031	0.767
Satisfied	0.009	0.642	0.000	1	0.988	1.009	0.287	3.556
**Work–life integration**								
Dissatisfied			3.470	2	0.176			
Neither	−0.604	0.605	0.997	1	0.318	0.547	0.167	1.789
Satisfied	−1.294	0.698	3.435	1	0.064	0.274	0.070	1.077
**Efficiency and resources**								
Dissatisfied			0.757	2	0.685			
Neither	0.136	0.602	0.051	1	0.821	1.146	0.352	3.730
Satisfied	−0.363	0.676	0.288	1	0.592	0.696	0.185	2.619
**Overall, I am satisfied with pursuing a career in medicine.**								
Disagree			1.900	2	0.387			
Neither agree/disagree	1.087	1.043	1.087	1	0.297	2.966	0.384	22.897
Agree	−0.007	0.921	0.000	1	0.994	0.993	0.163	6.042
**In general, I find my colleagues to be supportive.**								
Disagree			2.419	2	0.298			
Neither agree/disagree	−1.422	1.245	1.303	1	0.254	0.241	0.021	2.771
Agree	−0.003	1.127	0.000	1	0.998	0.997	0.110	9.082
**People treat each other with respect in my work group.**								
Disagree			1.072	2	0.585			
Neither agree/disagree	−0.616	1.485	0.172	1	0.678	0.540	0.029	9.928
Agree	0.340	1.345	0.064	1	0.801	1.404	0.101	19.616
**How well do you feel supported by your social support/friends/family?**								
Very well			7.599	2	**0.022**			
Neutral	1.537	0.733	4.397	1	**0.036**	4.651	1.106	19.568
Somewhat poorly	2.411	1.163	4.298	1	**0.038**	11.140	1.141	108.80
**How would you best describe what would occur if you reached out for help to those in your learning and work environment?**								
Very supportive			1.270	2	0.530			
Neutral	0.477	0.618	0.596	1	0.440	1.612	0.480	5.412
Somewhat poorly	1.009	1.140	0.783	1	0.376	2.742	0.294	25.590
**Do you feel that your medical school has enough strategies aimed at medical students’ well-being in place?**								
No			1.734	2	0.420			
Somewhat	−0.767	0.844	1.056	1	0.304	0.420	0.080	3.196
Yes	0.132	1.168	0.013	1	0.910	1.141	0.116	14.246
**Constant**	0.500	1.346	0.138	1	0.710	1.650		

## Data Availability

The data that support the findings of this study are available from the corresponding author, Vincent Agyapong, upon reasonable request.

## Appendix A. Medical Students Depression Survey Questions

Demographics:

1. What is your age?
  (a) <25
  (b) 25 to 30
  (c) 31–40
  (d) 40–60
  (e) >60
2. What is your gender
  (a) M
  (b) F
  (c) Other
3. Do you have any dependents? Check all that apply.
  - Children/Parents/Grandparents/Other
4. What best describes your current relationship status?
  - Married/Common-law/Committed relationship/Dating/Single/Other
5. How would you best identify your ethnicity?
  - Caucasian/Asian/Indigenous/Black/Other, please specify
6. How would you describe yourself in terms of your medical undergraduate training?
  - Canadian Medical Graduate/International Medical Graduate/Other
7. Which year of Medical School are you currently in? (check all that apply)
  (a) 1st year
  (b) 2nd year
  (c) 3rd year
  (d) 4th year
  (e) Clerkship
8. What residency program are you hoping to be part of after medical school?
  - Anesthesiology/General Surgery/Neurosurgery/Obstetrics and Gynecology/Ophthalmology/Orthopedic Surgery/Otolaryngology/Plastic Surgery/Urology/Vascular Surgery/Pathology/Dermatology/Radiology/Emergency/Family/Internal/Medical Genetics/Medical Microbiology/Neurology/Pediatric Neurology/Nuclear Medicine/Pediatrics/Physical Medicine and Rehabilitation/Psychiatry/Public Health and Preventative Medicine/Radiation Oncology
9. Please rate your degree of satisfaction with each of the following workplace dimensions during your clinical rotations.
  	**Very Dissatisfied**	**Dissatisfied**	**Neither**	**Satisfied**	**Very Satisfied**
  Quality of peer collaboration among your medical student colleagues					
  Quality of interaction with your attending physicians and residents					
  Quality of your learning environment					
  Workload and job demands					
  Control and flexibility					
  Work–life integration (meeting personal and professional obligations)					
  Efficiency and resources					
10. To what extent do you agree with the following statements?
  	**Strongly Disagree**	**Disagree**	**Neither Agree nor Disagree**	**Agree**	**Strongly Agree**
  Overall, I am satisfied about pursuing a career in medicine					
  In general, I find my colleagues to be supportive					
  People treat each other with respect in my work group					
  A spirit of cooperation and teamwork exists in my work group					
  Disputes or conflicts are resolved fairly in my work group					
11. Over the last 2 weeks, how often have you been bothered by any of the following problems?
  	**Not at All = 0**	**Several Days = 1**	**More than Half the Days = 2**	**Nearly Every Day = 3**
  Little interest or pleasure in doing things				
  Feeling down, depressed, or hopeless				
  Trouble falling or staying asleep, or sleeping too much				
  Feeling tired or having little energy				
  Poor appetite or overeating				
  Feeling bad about yourself or that you are a failure or have let yourself or your family down				
  Trouble concentrating on things such as reading the newspaper or watching television				
  Moving or speaking so slowly that other people could have noticed? Or the opposite, being so fidgety or restless that you have been moving around a lot more than usual				
  Thoughts that you would be better off dead or of hurting yourself in some way				
12. How well do you feel supported by your social supports/friends/family?
  - Very well/Somewhat well/Neutral/Somewhat poorly/Very poorly
13. How would you best describe what would occur if you reached out for help to those in your learning and work environment?
  - Very supportive/Somewhat supportive/Neutral/Somewhat hostile/Hostile
14. Do you feel that your medical school has enough strategies aimed at medical students’ well-being in place?
  - Yes/Somewhat/No
